# Investigating the role of the built environment, socio-economic status, and lifestyle factors in the prevalence of chronic diseases in Mashhad: PLS-SEM model

**DOI:** 10.3389/fpubh.2024.1358423

**Published:** 2024-05-15

**Authors:** Kiyavash Irankhah, Soheil Asadimehr, Behzad Kiani, Jamshid Jamali, Reza Rezvani, Seyyed Reza Sobhani

**Affiliations:** ^1^Department of Nutrition, Faculty of Medicine, Mashhad University of Medical Sciences, Mashhad, Iran; ^2^Social Determinants of Health Research Center, Mashhad University of Medical Sciences, Mashhad, Iran; ^3^UQ Center for Clinical Research, The University of Queensland, Brisbane, QLD, Australia; ^4^Department of Biostatistics, School of Health, Social Determinants of Health Research Center, Mashhad University of Medical Sciences, Mashhad, Iran

**Keywords:** chronic diseases, built environment, socio-economic status, food intake, physical activity

## Abstract

**Background:**

Chronic diseases remain a significant contributor to both mortality and disability in our modern world. Physical inactivity and an unhealthy diet are recognized as significant behavioral risk factors for chronic diseases, which can be influenced by the built environment and socio-economic status (SES). This study aims to investigate the relationship between the built environment, SES, and lifestyle factors with chronic diseases.

**Methods:**

The current study was conducted in Mashhad’s Persian cohort, which included employees from Mashhad University of Medical Sciences (MUMS). In the study, 5,357 participants from the cohort were included. To assess the state of the built environment in Mashhad, a Geographic Information System (GIS) map was created for the city and participants in the Persian Mashhad study. Food intake and physical exercise were used to assess lifestyle. A food frequency questionnaire (FFQ) was used to assess food intake. To assess food intake, the diet quality index was computed. To assess the link between variables, the structural model was created in accordance with the study’s objectives, and partial least square structural equation modeling (PLS-SEM) was utilized.

**Results:**

The chronic diseases were positively associated with male sex (*p* < 0.001), married (*p* < 0.001), and higher age (*p* < 0.001). The chronic diseases were negatively associated with larger family size (*p* < 0.05), higher SES (*p* < 0.001), and higher diet quality index (DQI) (*p* < 0.001). No significant relationship was found between chronic disease and physical activity.

**Conclusion:**

Food intake and socioeconomic status have a direct impact on the prevalence of chronic diseases. It seems that in order to reduce the prevalence of chronic diseases, increasing economic access, reducing the class gap and increasing literacy and awareness should be emphasized, and in the next step, emphasis should be placed on the built environment.

## Introduction

Globally, chronic illnesses are a leading source of mortality and disability. Cardiovascular disorders (heart attacks and strokes), cancers, chronic respiratory disorders, diabetes, chronic liver and kidney problems, and obesity are a few examples of chronic diseases (1, 2). These are responsible for 42 million deaths worldwide each year and for a large share of the disease’s burden (1, 3). Premature fatalities are defined as those that occur before the age of 70, such as the 17 million deaths caused by chronic diseases (4). In low- and middle-income nations, mortality from chronic diseases accounts for over three-quarters of all deaths (5, 6). According to the DALY index, 74% of deaths globally in 2019 were caused by chronic diseases, which also accounted for 64% of the global disease burden (7).

The identification of primary risk factors and population-level management of these disorders are essential components of chronic disease prevention (8). The four primary behavioral risk factors for chronic disease are alcohol use, smoking, poor diet, and inactivity. Blood lipid disorders, obesity, high blood pressure, and excessive blood sugar are the four biological risk factors. The emergence and aggravation of metabolic and physiological risk factors are significantly influenced by behavioral risk factors. But modifying these lifestyle choices can assist in managing these risk factors and reducing the burden of disease (9).

Excess energy consumption from high-calorie and low-nutrient density products (10), high consumption of sugar (11), sodium (12) and trans fatty acids (13), and poor consumption of dietary fiber (14), omega-3 (13), fruits and vegetables (15) all raise the risk of chronic diseases. Physical activity can lower the risk of chronic diseases by improving obesity and weight loss and strengthening the body’s antioxidant systems (16). People with lower socioeconomic level are also more vulnerable to chronic disease risk factors (17). Aside from an individual’s economic status, the built environment and physical characteristics of their residences can influence physical activity and food intake, both of which are factors that influence chronic diseases (18).

Chronic diseases currently place the greatest strain on Iran’s health-care system. According to WHO statistics, chronic diseases caused 82% of deaths in Iran in 2020 (43% cardiovascular diseases, 16% cancers, and 23% other) (19). Physical inactivity increased among Iranian adults from 39% in 2011 to 51.3% in 2021 (20–22). Every year, over 330,000 Iranians relocate to cities, which increases the need for basic infrastructure (23, 24). One of the factors influencing the rise in the prevalence of chronic diseases as a result of lifestyle changes is urbanization (1). Sedentary lifestyles and restricted availability to fresh meals are frequently associated with urbanization, particularly in less developed nations (25–27).

Iranians consume a diet low in protein, vegetables, and fruits and high in carbohydrates, especially bread and rice. In cities, there has also been a rise in the consumption of fast food and unhealthy snacks (28). The average salt consumption in the population is 9.52 grams per day, roughly double the World Health Organization guideline (29). Furthermore, in 2020, Iran’s *per capita* sugar consumption was 3.5 times higher than the advised level (30, 31). Among Iranian adults, highly processed foods account for over half of their daily calorie consumption (32). Examining these elements as a whole is essential given the established links between socioeconomic position, built environment, lifestyle and chronic diseases (28).

Although these relationships have been studied previously, there is still a lack of research on the combined effect of these connections on chronic diseases, especially in Iran and more specifically in Mashhad, the country’s second-largest city. Therefore, the main goal of this research is to clarify the complex connections between the built environment, diet, socioeconomic status, physical activity, and chronic diseases in Mashhad University of Medical Sciences staff members. Our goal is to obtain a thorough understanding of the factors that contribute to chronic diseases by investigating these interconnected aspects. This information will be useful in developing preventive strategies and health interventions.

## Methods

### Data and sample size

This cross-sectional study was conducted in the Mashhad Persian cohort study, which included Iranian citizens who were employed by Mashhad University of Medical Sciences, lived in Mashhad, and were between the ages of 30 and 70.

Each participant in the Persian Cohort Center provides written informed permission, which is obtained through the use of reliable personal identity documents. Samples of biological material are collected after registration, since participants are required to arrive fasting. The measurement of anthropometric traits comes next. Following the interview, the participants fill out three questionnaires on general health, medicine, and diet (29).

Based on a prevalence rate of 51.3%, a response rate of 90%, and a design effect of 1.5, a total sample size of 4,266 was calculated (22). Given that a larger sample size improves the accuracy of PLS-SEM estimations (30), we chose a sample size of 5,357 participants from the available Mashhad Persian cohort for the investigation. The research ethics committee of Mashhad University of Medical Sciences accepted this work with the number IR.MUMS.fm.REC.1396.620.

### Exposure measurement

Height (in centimeters), weight (in kilograms), waist, hip and wrist circumferences (in centimeters) were measured using Seca meters and scales following the protocols of the US National Institutes of Health (31).

The general questionnaire yielded the variables of age, gender, education, marital status, and number of family members. Furthermore, physical activity was assessed using a generic questionnaire. Participants’ physical activity was measured in MET-h/day (29). The general questionnaire looks at demographics, socioeconomic situation, occupational status, and exercise levels. This questionnaire is filled out by experts.

The wealth score index (WSI) and education (32) were used to assess socioeconomic position. The wealth score index (WSI) for each person was calculated using morphological component analysis (MCA) based on the following variables: [access to a freezer, access to a washing machine, access to a dishwasher, access to a computer, access to the Internet access to a motorcycle, access to a car (no access, access to a car with a price of <50 million Tomans and access to a car with a price of >50 million Tomans), access to a vacuum cleaner, type of color TV (black and white TV or color TV) regular vs. plasma color TV], owning a mobile phone, owning a computer or laptop, lifetime international travel (never, pilgrimage only, both pilgrimage and non-pilgrimage).

Nutritionists are trained to complete the nutrition questionnaire. This questionnaire looks at meal frequency, eating patterns, food preparation, and storage practices. The diet quality index (DQI) and a 134-part semi-quantitative food frequency questionnaire (FFQ) (33) which ask about the amount of people’s usual consumption of each food during the year before the date of the interview, were used to determine food consumption. Participants reported their daily, weekly, monthly or yearly use of each item, as well as the portion consumed each time, based on portion sizes pertaining to each item. Actual dishes, cups and utensils, as well as several portion size models were used for a more precise portion size estimation. In addition, a 64-picture album including standard portions for selected items was used whenever needed (34). The Diet Quality Index is a technique for assessing the overall quality of a person’s dietary intake by grading food or nutrients, as well as lifestyle, in accordance with existing nutritional guidelines. This index’s key components are diversity, sufficiency, moderation, and balance, which were calculated separately (35). Diet quality index, a higher score indicates a higher diet quality.

Food diversity considers the intake of five essential food groups: cereals, vegetables, fruits, dairy and legumes, meats. Each group consumed earns 3 points, resulting in a maximum diversity score of 15 and a minimum of zero. Adequacy evaluates the consumption of eight vital food items, such as vegetables, fruits, grains, fiber, protein, iron, calcium, and vitamin C. Scores range from 0 to 5 based on the percentage of daily requirements, yielding a maximum adequacy score of 40 and a minimum of zero. Dietary adequacy assesses five items including total fat, saturated fat, cholesterol, sodium, and foods with minimal nutritional value. Scores range from 0 to 6, reflecting the percentage of daily recommended intake. The maximum suitability score is 30, while the minimum is zero. Nutritional balance analyzes macronutrient and fatty acid ratios, with scores ranging from 0 to 6 and 0 to 4, respectively. This results in a maximum balance score of 10 and a minimum of zero (35).

Population density, land use mix, access to walking space and pavement area, access to public transportation, area of roads and main intersections, and access to various types of shops and restaurants such as supermarkets, bakeries, fruit and vegetable shops, fast food outlets, coffee shops, and grills were all used to evaluate the built environment. Each feature was measured in proportion to the area of the neighborhood, the data and number of the variables were obtained from municipal maps. Mashhad has 175 neighborhoods, according to the municipality’s urban division. The map was then produced using a geographic information system (GIS).

### Outcome measurement

Medical history, reproductive history (women), medication history, family medical history, oral and dental health (past and current), personal habits (smoking, alcohol and drug use) (past and current), blood pressure and pulse measurement, and physical examination are all examined. The physician completes this questionnaire, and the ailments of the participants are diagnosed by the physician. Chronic diseases included cardiovascular disease, diabetes, cancer, chronic renal disease, liver disease, lung disease, and obesity. Chronic patients were participants who had at least one of these disorders (29).

### Statistical analysis

SPSS 26 was used for data analysis, and ArcGIS 10.6 was used to examine the built environment. We also used Smart PLS 3.2.8 to simulate partial least squares structural equations. The data was analyzed using descriptive statistics such as frequency, mean, and standard deviation. For comparing quantitative variables with normal distributions, the independent t-test was used, and for comparing quantitative variables with non-normal distributions, the Mann–Whitney test was utilized. To compare qualitative variables, the chi-square test was performed. The mean and standard deviation were used to convey quantitative data, whereas frequencies and percentages were used to express qualitative data. To examine the link between latent variables, partial least squares structural equation modeling (PLS-SEM) was utilized.

The bootstrapping algorithm was used to examine the significance of the association between possible variables. The coefficient of determination (R2), path coefficient, and effect size (f2) were all evaluated as part of the internal model quality study. R2 was used to assess the structural model’s explanatory and predictive capacity, with values ranging from 0 to 1. R2 values of 0.19, 0.33, and 0.67 were proposed by Urbach and Ahleman for small, medium, and strong explanatory powers, respectively. Cohen’s suggested f2 values for weak, medium, and strong effects are 0.02, 0.15, and 0.35, respectively. Path coefficient values range from −1 to +1, with a coefficient closer to 1 suggesting a stronger positive or negative connection (30, 36).

## Results

In our current study, we enrolled 5,357 participants from the Mashhad Persian cohort study. Among them, 2,413 were men (45%) and 2,944 were women (55%). Within this cohort, 2,247 individuals had chronic diseases, while 3,110 were in good health. Table 1 provides a summary of key findings: the average age of the participants was 43.76 ± 8.00 years, and each participant had an average of 4 family members. Additionally, 4,586 people were married, while 771 people were single. From a socio-economic perspective, 4,408 participants held a university education, 680 individuals had a diploma, 262 people had education below a diploma, and only 10 individuals were illiterate. The average wealth index in the studied population was −0.01 ± 0.98. In addition, in this table, information on the participants’ physical activity and food intake is provided.

**Table 1:** Data characteristics: chronic disease and healthy participants.

Variables (n)		Total (5,357)	Chronic disease (2,247)	Healthy participants (3,110)	*p*-value
Gender	Men [*n* (%)]	2,413 (45%)	1,052 (44%)	1,361 (56%)	<0.05
	Women [*n* (%)]	2,944 (55%)	1,195 (41%)	1749 (59%)	
Age (mean years)		43.76 ± 8.00	45.94 ± 8.93	42.19 ± 6.83	<0.001
Family size (mean number)		3.44 ± 1.06	3.45 ± 1.05	3.43 ± 1.05	0.64
Marital	Single [*n* (%)]	771 (14%)	262 (34%)	509 (66%)	<0.001
	Married [*n* (%)]	4,586 (86%)	1985 (43%)	2,601 (57%)	
Social economic					
Education	Illiterate [*n* (%)]	10 (0.18%)	7 (70%)	3 (30%)	<0.001
	Under diploma [*n* (%)]	262 (5%)	161 (61%)	110 (39%)	
	Diploma [*n* (%)]	680 (13%)	335 (49%)	345 (51%)	
	University [*n* (%)]	4,405 (82%)	1744 (40%)	2,661 (60%)	
Wealth index		−0.01 ± 0.98	−0.02 ± 1.00	−0.01 ± 0.97	0.83
Physical activity					
Physical activity (MET-h/day)		38.86 ± 5.57	38.50 ± 6.06	39.11 ± 5.51	<0.001
Food intake					
Energy (Cal/day)		2414.62 ± 673.26	2425.34 ± 682.32	2408.01 ± 664.05	0.33
Cereals (gr/day)		468.91 ± 358.52	473.31 ± 364.30	465.64 ± 355.82	0.44
Vegetables (gr/day)		367.79 ± 194.91	388.00 ± 204.57	353.16 ± 186.31	<0.001
Fruits (gr/day)		456.35 ± 277.32	486.52 ± 296.44	434.57 ± 260.55	<0.001
Dairy (gr/day)		387.12 ± 233.78	394.81 ± 241.48	381.53 ± 227.96	<0.05
Legumes and nuts (gr/day)		63.18 ± 44.29	63.85 ± 43.34	62.69 ± 44.98	0.34
Meats (gr/day)		116.98 ± 57.93	116.20 ± 57.59	117.52 ± 58.18	0.41
Sugars (gr/day)		134.12 ± 115.74	133.29 ± 121.08	134.69 ± 111.74	0.66
Fats (gr/day)		27.78 ± 19.30	26.58 ± 18.24	28.81 ± 19.99	<0.001
Varity		14.91 ± 2.76	14.89 ± 2.78	14.92 ± 2.74	0.73
Adequacy		26.97 ± 4.95	27.10 ± 4.85	26.87 ± 5.03	0.11
Moderation		13.50 ± 5.95	13.88 ± 6.14	13.32 ± 5.80	<0.001
Balance		0.49 ± 1.00	0.51 ± 1.01	0.48 ± 0.99	0.27
Diet quality index		55.86 ± 7.71	56.37 ± 7.82	55.50 ± 7.61	<0.001

Quantitative variables, values are reported as mean ± standard deviation and qualitative variables are reported as number (percentage). Independent *t*-test and chi-square test were used to compare variables between two groups.

Between healthy participants and chronic illness groups, demographic and socioeconomic factors related to physical activity and food consumption were evaluated. When compared to the chronic disease group, the healthy group had a considerably higher number of female participants, a higher number of single individuals, a higher education level, a higher average physical activity, and a higher consumption of fat. In comparison to the healthy group, the chronic illness group had a considerably larger percentage of illiterates, a higher average age, a poorer diet quality score, lower moderation, and lower consumption of vegetables, fruits, and dairy products.

Table 2 provides insights into the environmental access and food availability for participants within their neighborhood. The differences in built environment features between the two groups were explored (Table 2). The chronic disease group had considerably higher values for land use mix, pedestrian bridges ratio, access to supermarkets ratio, and access to coffee shop juice ratio.

**Table 2:** Environmental access and food access separately from the chronic disease and healthy participants.

Variables (n)	Total (5,357)	Chronic disease (2,247)	Healthy participants (3,110)	*P*-value
*Environmental access*				
Land use mix	0.0022 ± 0.00	0.0022 ± 0.00	0.0021 ± 0.00	<0.05
Open space ratio	0.43 ± 1.28	0.45 ± 1.32	0.42 ± 1.25	0.51
Public transportation supply	39.86 ± 51.12	40.51 ± 50.40	39.41 ± 51.65	0.44
Non-motorized transport	181.60 ± 56.76	182.98 ± 55.71	181.63 ± 57.43	0.14
Road area ratio	29.46 ± 7.80	29.60 ± 7.70	29.36 ± 7.87	0.29
Sidewalk area ratio	8.68 ± 7.43	8.60 ± 6.83	8.73 ± 7.84	0.51
Pavement area ratio	588.19 ± 804.48	594.29 ± 861.55	583.68 ± 760.96	0.64
Main road intersection ratio	15.39 ± 17.84	15.53 ± 17.59	15.30 ± 18.02	0.66
Pedestrian bridges ratio	2.17 ± 2.34	2.26 ± 2.41	2.01 ± 2.28	<0.05
*Food access*				
Access to restaurants	7.63 ± 4.70	7.78 ± 4.72	7.53 ± 4.68	0.06
Access to supermarkets	7.97 ± 4.40	8.12 ± 4.45	7.86 ± 4.36	< 0.05
Access to fast food	8.71 ± 5.79	8.69 ± 5.66	8.72 ± 5.88	0.86
Access to fruit and vegetables	5.58 ± 3.16	5.62 ± 3.16	5.54 ± 3.16	0.36
Access to bakery	4.46 ± 2.93	4.45 ± 2.96	4.47 ± 2.92	0.77
Access to coffee shop	6.37 ± 5.40	6.57 ± 5.40	6.23 ± 5.39	< 0.05
Access to grill	5.02 ± 3.30	5.02 ± 3.25	5.02 ± 3.33	0.99

Quantitative variables, values are reported as mean ± standard deviation. Independent *t*-test was used to compare variables between two groups.

**Figure 1:**
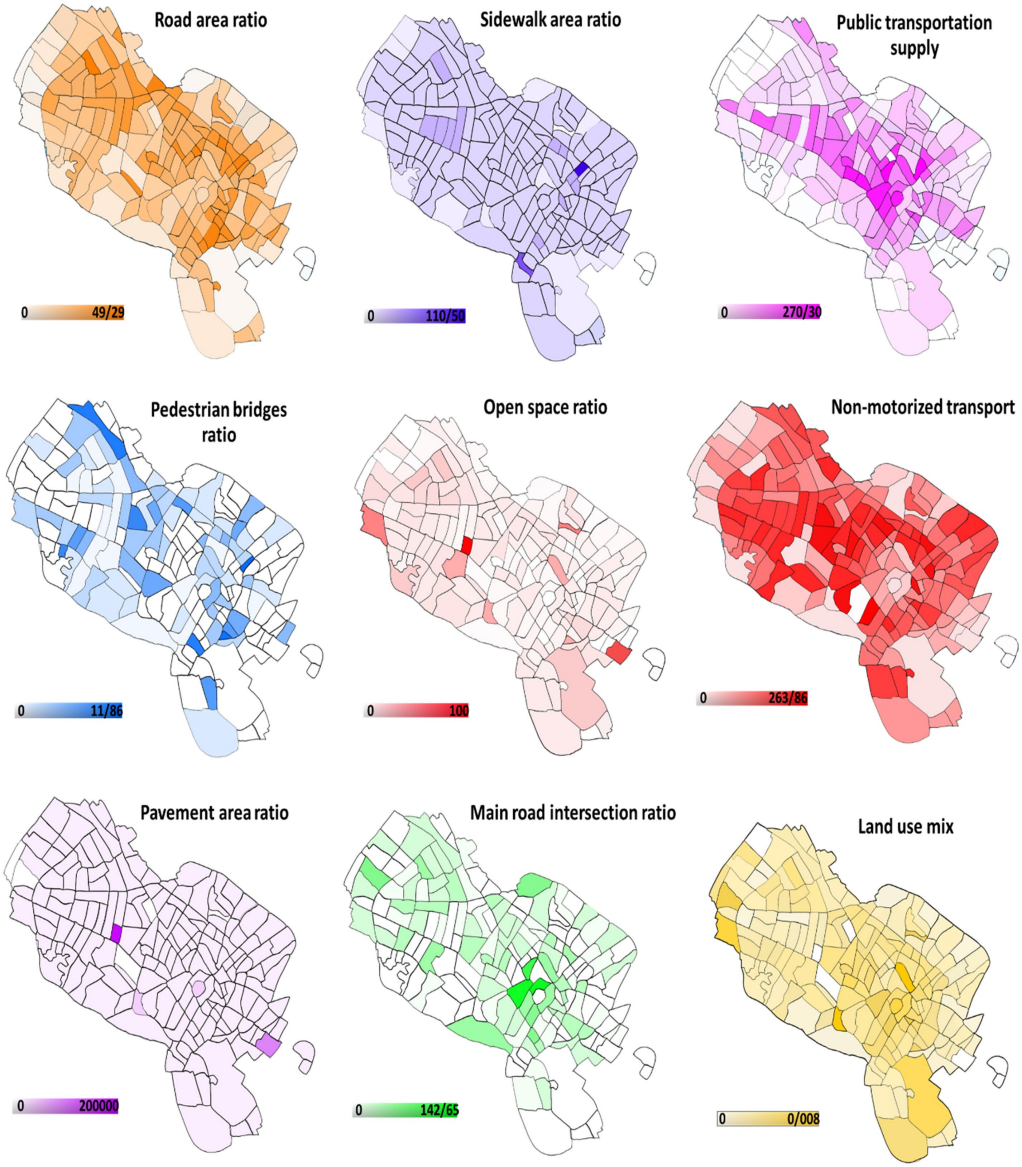
The environmental access map of Mashhad by neighborhoods.

Figure 1 depicts Mashhad’s environmental access map by neighborhood. In comparison to other places, the western areas and the city center have more environmental accessibility.

**Figure 2:**
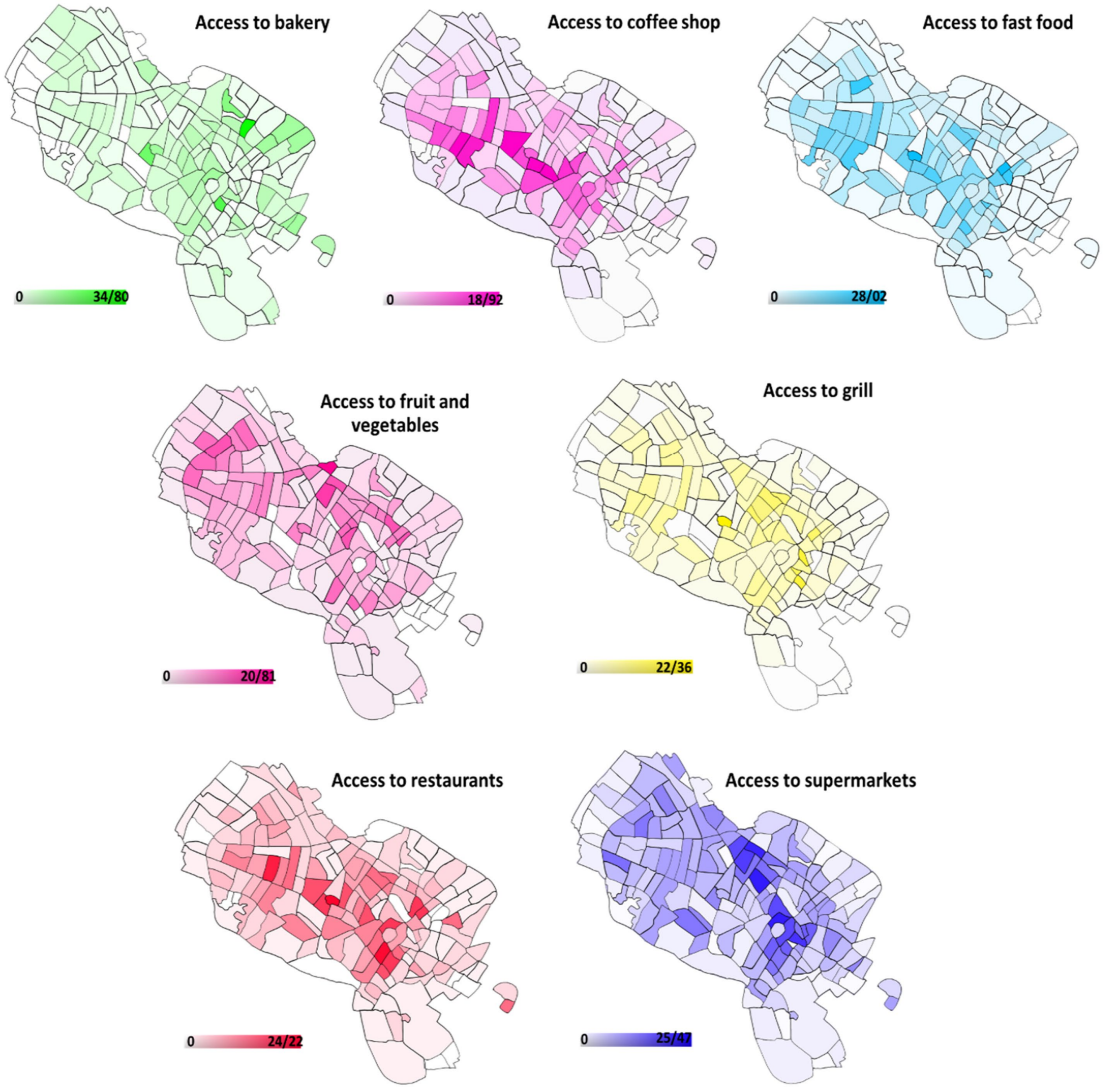
The food access map of Mashhad by neighborhoods.

Figure 2 depicts Mashhad’s food access map by neighborhood. Restaurants, fruits and vegetables, fast food, and juice were more readily available in the western regions. Northern areas had greater access to bakeries. Supermarkets and grills were more accessible in central regions.

Table 3 shows the bivariate association patterns of the built environment, socioeconomic status, diet quality index, and physical activity with the state of chronic diseases (hidden variables). Chronic disease was significantly associated with married persons, higher environmental access, higher access to unhealthy food stores, and older age, and adversely associated with higher socioeconomic position, higher diet quality index, and higher number of family members.

**Table 3:** Descriptive analysis for the relationship between built environment, socioeconomic status, diet quality index and physical activity with chronic disease status: bivariate correlation.

latent variable	SES	Chronic disease	Marital status	Family size	Gender	DQI	Environmental access	Age	Healthy food stores	Unhealthy food stores	P. A
SES	1	−0.195^**^	0.001	0.044^**^	0.122^**^	0.001	0.089^**^	−0.320^**^	0.075^**^	0.049^**^	−0.016
Chronic disease		1	0.029^*^	−0.068^**^	−0.001	−0.054^**^	0.029^*^	0.352^**^	0.016	0.025^*^	−0.003
Marital status			1	0.201^**^	−0.245^**^	0.018	0.005	0.024^*^	0.000	0.000	−0.025^*^
Family size				1	−0.065^**^	0.023	−0.031^**^	−0.098^**^	−0.004	−0.028^*^	0.018
Gender					1	−0.063^**^	−0.013	−0.127^**^	0.024^*^	−0.007	−0.011
DQI						1	−0.026^*^	−0.073^**^	−0.002	−0.003	0.019
Environmental access							1	0.093^**^	0.276^**^	0.207^**^	−0.009
Age								1	0.040^**^	0.031^**^	−0.010
Healthy food stores									1	0.256^**^	0.005
Unhealthy food stores										1	0.007
P. A											1

^**^The correlation is significant at the 0.01 level. ^*^Correlation is significant at the 0.05 level. Correlations between quantitative variables were calculated using Pearson correlation, while the correlation between a binary nominal variable and a continuous variable was calculated using the Point-Biserial Correlation test.

**Figure 3:**
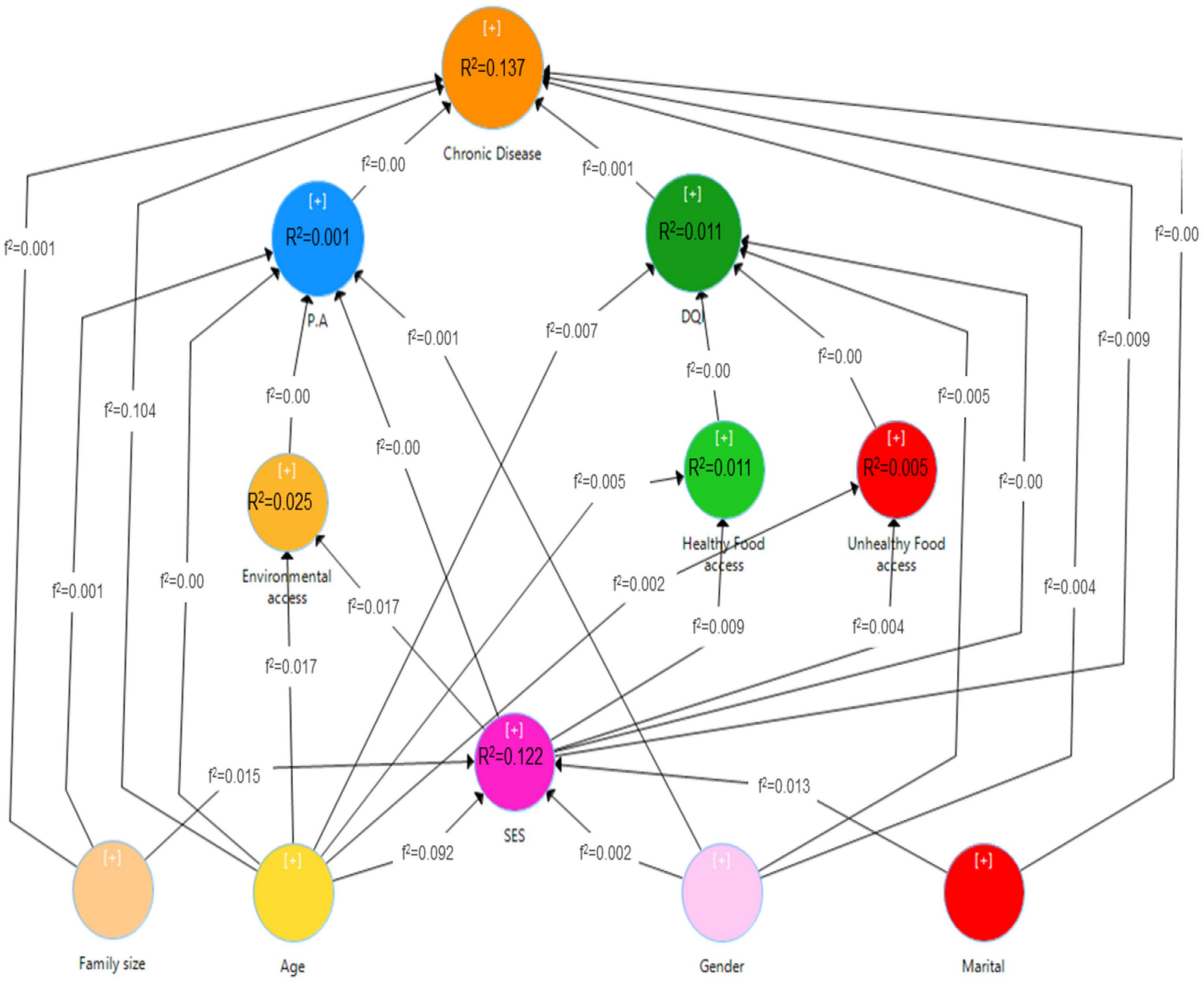
Path analysis model of the relationship between built environment, socio-economic status, diet quality index and physical activity with the status of chronic diseases.

The combined model is depicted in Figure 3. There was a positive relationship between chronic disease and older age [*b* (*SD*) = 0.322 (0.014), *p* < 0.001], male sex [*b* (*SD*) = 0.058 (0.013), *p* < 0.001], and married status [*b* (*SD*) = 0.044 (0.011), *p* < 0.001]. Higher diet quality index [*b* (*SD*) = −0.026 (0.012), *p* < 0.001], higher socioeconomic level [*b* (*SD*) = −0.097 (0.016), *p* < 0.001], and larger family size [*b* (*SD*) = −0.036 (0.012), *p* < 0.05] were found to have a negative correlation with chronic disease. Physical activity had no relationship with chronic illness.

Male sex [*b* (*SD*) = −0.072 (0.012), *p* < 0.001] and older age [*b* (*SD*) = −0.089 (0.014), *p* < 0.001] showed a negative link with a greater diet quality score, whereas socioeconomic level, availability to healthy food stores, and access to unhealthy food stores did not.

Physical activity was unrelated to gender, age, financial level, environmental accessibility, or family size.

Access to healthy food shops was positively related to older age [*b* (*SD*) = 0.071 (0.012), *p* < 0.001] and higher socioeconomic status [*b* (*SD*) = 0.098 (0.014), *p* < 0.001], whereas access to unhealthy food stores did not relate to age or socioeconomic status. Environmental accessibility was positively related to older age [*b* (*SD*) = 0.135 (0.021), *p* < 0.001] and better socioeconomic level [*b* (*SD*) = 0.132 (0.023), *p* < 0.001].

Male sex [*b* (*SD*) = 0.191 (0.011), *p* < 0.001] and married status [*b* (*SD*) = 0.028 (0.011), *p* < 0.05] were related to greater socioeconomic position. Finally, older age [*b* (*SD*) = −0.308 (0.013), *p* < 0.001] had a negative relationship with socioeconomic position, although family size was not.

Figure 3 depicts the coefficient of determination (R2), which reflects the predictive model’s strength in identifying factors influencing chronic diseases. This model could account for 13.7% of the variance in chronic disease, 1.1% in diet quality index, 0.1% in physical activity, 2.4% in environmental access, 1% in access to healthy food stores, 0.5% in access to unhealthy food stores, and 12.2% in economic status. The effect sizes (f2) for all variables in the model were small, with no evidence of strong or moderate impacts.

Table 4 shows the results of the indirect effects. The examination of indirect effects found that older age was associated with chronic disease indirectly (*β* = 0.032, *p* < 0.001) via food intake and socioeconomic status. Furthermore, older age was associated with better environmental access (*β* = −0.041, *P*<0.001) and greater availability to healthy food stores (*β* = −0.030, *p* < 0.001) via socioeconomic level. Through the diet quality index and socioeconomic level, male sex was found to be indirectly associated to chronic disease (*β* = −0.007, *p* < 0.001). Furthermore, through socioeconomic position, male sex was indirectly related to higher diet quality index (*β* = −0.002, *p* < 0.001), larger environmental access (*β* = 0.012, *p* < 0.001), and higher access to healthy food stores (*β* = 0.009, *p* < 0.001). Through socioeconomic level, married people were indirectly associated with chronic disease (*β* = −0.003, *P* < 0.05), more environmental access (*β* = 0.004, *p* < 0.05), and greater access to healthy food shops (*β* = 0.003, *p* < 0.05).

**Table 4:** The indirect effect of the relationship between the built environment, socio-economic status, diet quality index and physical activity with the status of chronic diseases.

Path	*β*	*P*-value	Relationships	Indirect effect		Total effect	
				*β*	*P*-value	*β*	*p*-value
Age → DQI → Chronic disease	0.002	0.04	Age → Chronic disease	0.032	0.000	0.354	<0.001
Age → SES → Chronic disease	0.030	<0.001					
Age → SES → DQI	0.006	0.11	Age → DQI	0.006	0.114	−0.083	<0.001
Age → SES → Environmental access	−0.041	<0.001	Age → Environmental access	−0.041	0.000	0.094	<0.001
Age → SES → Healthy food stores	−0.030	<0.001	Age → Healthy food stores	−0.030	0.000	0.041	<0.001
			Age → SES			−0.308	<0.001
			DQI → Chronic disease			−0.026	0.03
Family size → SES → Chronic disease	−0.001	0.25	Family size → Chronic disease	−0.001	0.268	−0.037	0.001
Gender → DQI → Chronic disease	0.002	0.06	Gender → Chronic disease	−0.007	0.001	0.051	<0.001
Gender → SES → Chronic disease	−0.009	<0.001					
Gender → SES → DQI	−0.002	0.12	Gender → DQI	−0.002	0.123	−0.074	<0.001
Gender → SES → Environmental access	0.012	<0.001	Gender → Environmental access	0.012	0.000	0.012	<0.001
Gender → SES → Healthy food stores	0.009	<0.001	Gender → Healthy food stores	0.009	0.000	0.009	<0.001
			Gender → SES			0.091	<0.001
Marital status → SES → Chronic disease	−0.003	0.02	Marital status → Chronic disease	−0.003	0.024	−0.003	<0.001
Marital status → SES → Environmental access	0.004	0.02	Marital status → Environmental access	0.004	0.022	0.004	0.02
Marital status → SES → Healthy food stores	0.003	0.02	Marital status → Healthy food stores	0.003	0.023	0.003	0.02
			Marital status → SES			0.028	0.01
			SES → Chronic disease	0.000	0.335	−0.097	<0.001
			SES → Environmental access			0.132	<0.001
			SES → Healthy food stores			0.098	<0.001

The indirect effect of the relationship between the variables was measured using PLS-SEM. A significance level of *P* < 0.05 was considered.

## Discussion

This cross-sectional study delves into the intricate interplay among chronic diseases, the built environment, socioeconomic status, dietary habits, and physical activity, employing a structural equation model. Highlighting the pivotal role of individual behaviors like physical activity and diet in chronic disease development, the model underscores the profound impact of the built environment on health outcomes. Furthermore, it sheds light on how socioeconomic status influences both the environment and lifestyle choices. The study’s findings reveal a higher incidence of chronic diseases among men, married individuals, and the older adult, contrasted with a lower prevalence among those from larger families, higher socioeconomic strata, and with healthier dietary habits.

According to the current study, higher diet quality is inversely connected to the risk of chronic diseases. Our findings are comparable with those of Hlaing et al. (37), who conducted research in Australia. According to this study, improved diet quality is inversely related to chronic disease outcomes in middle-aged Australian women. Furthermore, the findings of our study are consistent with the findings of Imelda Angeles et al. (38), in the Philippines, who found that consuming meat, sugary drinks, rice, and fish is connected with an increased risk of cardiovascular disease. Excessive calorie intake, processed meats, sugary drinks, refined carbohydrates, sugars, and fats can all raise the risk of chronic diseases (39, 40). Excessive consumption of simple carbohydrates, refined grains, saturated and trans fats, and sodium can result in dyslipidemia, hypertension, insulin resistance, and endothelial dysfunction (41–44). Mashhad’s culinary culture is rich and diverse, reflecting the broader Iranian cuisine. Traditional Iranian dishes are often centered around rice, bread and meat. However, with modernization and urbanization, there has been a shift toward more processed and fast foods, which are often high in unhealthy fats, sugars, and sodium.

According to the findings of this study, socioeconomic level has a negative association with the chance of getting chronic diseases. The findings are congruent with those of Shams et al. (8), Emami et al. (45), Moradi et al. (46), and Yaya et al. (47). A higher socioeconomic level provides more access to open spaces, physical activity facilities, and healthy food options, which leads to increased physical activity and improved diet quality, lowering the risk of chronic diseases (48, 49). Conversely, low socioeconomic status is associated with increased inactivity, smoking, alcohol consumption and unhealthy food choices, leading to an increased risk of chronic diseases (50–52). Lower socioeconomic status is also linked to poor education, insufficient nutrition knowledge, poor access to healthcare and seeking care at non-curable stages of the disease (53). Spending on cigarettes, tobacco and alcohol also reduces food budgets, depriving individuals of healthy and fresh foods (51).

The present study showed that physical activity is not related to the state of chronic diseases. The result obtained is contrary to most studies that state that physical activity and the chance of chronic diseases have an inverse relationship (54–56). Several variables could explain the ambiguous association between physical exercise and chronic diseases. The participants’ chronic conditions had already been diagnosed at the time of examination, and they had most likely received the appropriate instruction to change their lifestyle (29). The technique employed to assess physical activity may have been inaccurate. Furthermore, in the city of Mashhad, poor socioeconomic conditions can make it difficult for people to participate in physical activity programs or sports clubs due to cost or time constraints (57–59). Undesirable socioeconomic conditions can also result in psychological pressure and stress, lowering motivation and energy for physical exercise (60). Additionally, Mashhad’s status as a religious city in Iran introduces unique cultural and religious factors that could potentially hinder certain groups from engaging in physical activity, despite the presence of available facilities (61).

In the present study, no correlation was observed between the built environment and chronic diseases. The obtained result is contrary to most studies (62–64). Our findings are consistent with the findings of Maike Schulz et al. (65) in Germany, who investigated the influence of the built environment on risk factors and health behavior. The study found no association between green space or street design and health (65). In the context of Mashhad, several factors may contribute to this lack of correlation. Inadequate active transportation infrastructure, poor public transportation quality, and personal preference for personal autos over public transportation may have contributed to the lack of a link between the built environment and chronic diseases (66, 67). Additionally, the study focused on neighborhood facilities, assuming individuals used them, which may not always be the case. Personal preference, cost-effective grocery shopping outside the neighborhood and extensive marketing from stores outside the neighborhood may have influenced individuals’ facility use despite having access within the neighborhood (68, 69).

### Policy implication of findings

This study highlights the direct impact of socioeconomic status and dietary habits on the development of chronic diseases. Moreover, socioeconomic status significantly influences dietary choices. These findings underscore the importance of implementing targeted policies to mitigate the risk factors associated with chronic illnesses. By adopting these policy measures, governments and stakeholders can collaboratively strive to alleviate the burden of chronic diseases and foster healthier lifestyles within communities, thereby enhancing overall public health outcomes.

Improving Socioeconomic Conditions: Acknowledging the intricate connection between socioeconomic status and the risk of chronic diseases, elevating individuals’ socioeconomic status emerges as a pivotal strategy for preventing and mitigating chronic illnesses. Policy initiatives should concentrate on bridging socioeconomic disparities to facilitate equitable access to nutritious food, avenues for physical activity, and healthcare services. Initiatives aimed at poverty alleviation, educational enhancements, and fostering employment opportunities hold promise in fostering improved overall health outcomes (70–72).

Nutritional Education and Promotion: Given the inverse relationship between diet quality and chronic diseases, policymakers can prioritize nutritional education initiatives to improve food literacy and promote healthy eating habits. This could involve public awareness campaigns, nutritional education programs in schools, and community-based interventions aimed at increasing access to and knowledge of healthy food options (73–75).

Enhancing Food and Nutrition Literacy: Given the concerning findings indicating low levels of food and nutrition literacy in Iran (76, 77), it’s evident that many individuals, particularly those with low incomes, may lack access to accurate information regarding healthy eating habits and the correlation between diet and chronic diseases (53). To address this issue, policymakers must prioritize investments in initiatives aimed at enhancing nutritional knowledge and awareness among the populace. This may entail integrating comprehensive nutrition education into school curricula, establishing resources for nutritional counseling, and ensuring widespread access to reliable and precise nutritional information for the general public (78, 79).

Promoting Physical Activity: Despite the lack of correlation found in the study, physical activity remains a crucial factor in preventing chronic diseases. Policymakers can implement strategies to encourage physical activity, such as building and maintaining recreational facilities, providing incentives for active transportation, and promoting community-based exercise programs (80, 81).

Creating Healthy Built Environments: While the study did not find a direct association between the built environment and chronic diseases, policymakers can still prioritize urban planning strategies that promote active living. This could include designing walkable neighborhoods, enhancing public transportation infrastructure, and increasing access to green spaces for recreational activities (82, 83).

Utilizing Economic Incentives: Policymakers can leverage economic mechanisms such as subsidies and taxes to encourage healthy behaviors and discourage unhealthy ones. For example, subsidies could be provided for healthy food options, while taxes could be imposed on sugary beverages or unhealthy food products (84, 85).

### Strength and limitation

The study benefits from the utilization of a diverse range of influential variables for chronic diseases and employs partial least squares structural equation modeling, enhancing its robustness. However, the cross-sectional design poses limitations in establishing causal relationships between the built environment, socioeconomic characteristics, nutritional consumption, physical activity, and chronic diseases. Notably, the study lacks consideration of participants’ socio-economic status, including job, income, and housing status, which could offer a more comprehensive understanding of the socio-economic landscape. Moreover, behavioral and cultural factors were not accounted for in the study; their intricate roles in access and food choices warrant further exploration. Also, the data about diseases are self-reported.

Regarding the assessment of the built environment, municipal data was utilized, yet its potential limitations in comprehensiveness and currency should be acknowledged. This data may not be up-to-date and may not include all stores. Future studies may benefit from supplementing such data with questionnaires, interviews, or network buffers for a more nuanced evaluation. Additionally, it’s crucial to recognize that the study’s participant pool, limited to employees of Mashhad University of Medical Sciences, may not fully represent the broader Mashhad community.

Furthermore, the model’s explanatory power is modest, accounting for only 13.7% of chronic diseases. This underscores the need for additional research to explore the new links and factors influencing chronic diseases comprehensively.

## Conclusion

Cultural, environmental, economic, and lifestyle factors all have an impact on chronic diseases. The current study found that, in addition to its direct effect, socioeconomic position influences the prevalence of chronic diseases via its effect on food intake. It was also shown that nutrition is associated with the presence of chronic diseases. Policymakers and planners should create supportive economic, social, and cultural systems to prevent and minimize chronic diseases, such as by raising food and nutrition literacy, encouraging physical exercise, lowering poverty, and providing universal insurance coverage. These strategies have the potential to lower the prevalence of chronic diseases and improve community health.

## Data availability statement

The raw data supporting the conclusions of this article will be made available by the authors, without undue reservation.

## Ethics statement

The studies involving humans were approved by Mashhad University of Medical Sciences IR.MUMS.fm.REC.1396.620. The studies were conducted in accordance with the local legislation and institutional requirements. The participants provided their written informed consent to participate in this study.

## Author contributions

KI: Conceptualization, Data curation, Formal analysis, Investigation, Methodology, Software, Validation, Writing – original draft, Writing – review & editing. SA: Data curation, Investigation, Methodology, Software, Writing – original draft. BK: Conceptualization, Data curation, Formal analysis, Methodology, Software, Writing – review & editing. JJ: Formal analysis, Investigation, Methodology, Software, Writing – review & editing. RR: Funding acquisition, Project administration, Resources, Writing – review & editing. SS: Conceptualization, Data curation, Formal analysis, Funding acquisition, Investigation, Methodology, Project administration, Resources, Software, Supervision, Validation, Visualization, Writing – original draft, Writing – review & editing.
