# Supplementary material for: Investigating the role of the built environment, socio-economic status, and lifestyle factors in the prevalence of chronic diseases in Mashhad: PLS-SEM model
Source: Front Public Health. 2024 May 15;12:1358423. doi: 10.3389/fpubh.2024.1358423 (PMC11133713; doi:10.3389/fpubh.2024.1358423)
Supplement: Supplementary file 1 [file Data_Sheet_1.docx]

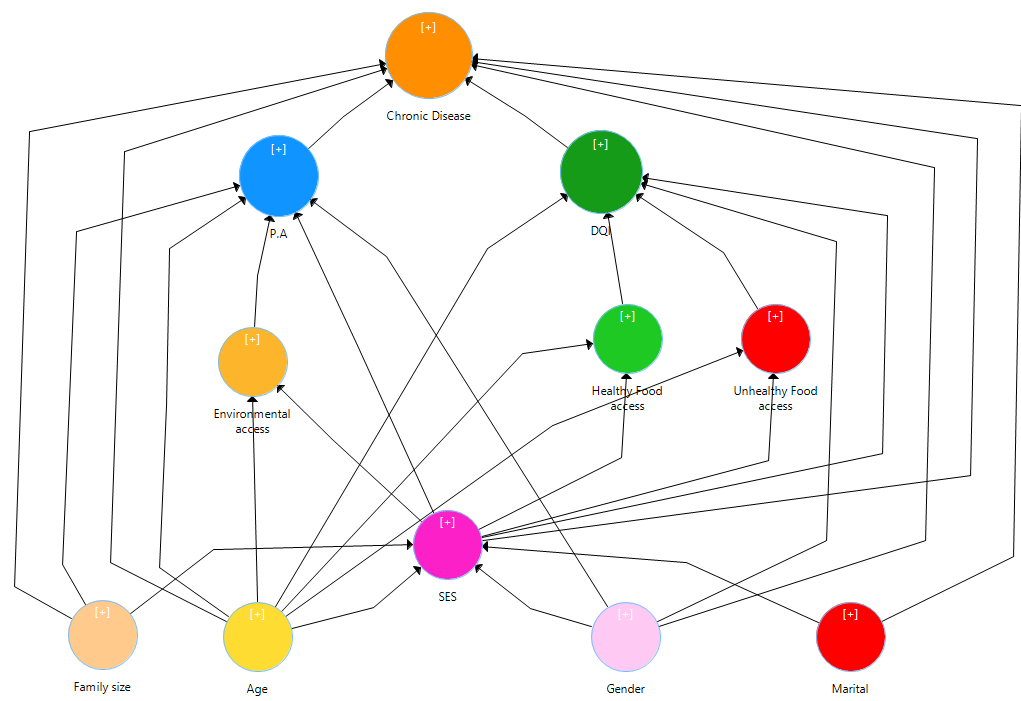


Appendix 1 Structural model of the relationship between built environment, socio-economic characteristics, life style factors with chronic diseases

Appendix 2 The results of analysis of the relationship between built environment, socio-economic status, diet quality index and physical activity with the status of chronic diseases

| **Relationship** | **Coefficient (SD)** | **T (P-Value)** |
| --- | --- | --- |
| Age **->** Chronic disease | 0.322 (0.014) | 23.627 (0.000) |
| Age -> DQI | -0.089 (0.014) | 6.541 (0.000) |
| Age -> Environmental access | 0.135 (0.021) | 6.410 (0.000) |
| Age -> Healthy food stores | 0.071 (0.012) | 6.013 (0.000) |
| Age -> P. A | -0.015 (0.013) | 1.158 (0.248) |
| Age -> SES | -0.308 (0.013) | 23.670 (0.000) |
| Age -> Unhealthy food stores | 0.052 (0.041) | 1.289 (0.198) |
| DQI -> Chronic disease | -0.026 (0.012) | 2.121 (0.034) |
| Environmental access -> P. A | -0.006 (0.013) | 0.439 (0.661) |
| Family size -> Chronic disease | -0.036 (0.012) | 3.091 (0.002) |
| Family size -> P. A | 0.017 (0.011) | 1.470 (0.141) |
| Family size -> SES | 0.014 (0.011) | 1.140 (0.229) |
| Gender -> Chronic disease | 0.058 (0.013) | 4.869 (0.000) |
| Gender -> P. A | -0.009 (0.011) | 0.774 (0.410) |
| Gender -> DQI | -0.072 (0.012) | 6.017 (0.000) |
| Gender -> SES | 0.091 (0.011) | 7.929 (0.000) |
| Healthy food stores -> DQI | 0.005 (0.013) | 0.380 (0.708) |
| Marital status -> Chronic disease | 0.044 (0.011) | 3.966 (0.000) |
| Marital status -> SES | 0.028 (0.011) | 2.623 (0.011) |
| P. A -> Chronic disease | 0.002 (0.011) | 0.161 (0.874) |
| SES -> Chronic disease | -0.097 (0.016) | 6.362 (0.000) |
| SES -> DQI | -0.019 (0.012) | 1.576 (0.110) |
| SES -> Environmental access | 0.132 (0.023) | 7.069 (0.000) |
| SES -> Healthy food stores | 0.098 (0.014) | 7.148 (0.000) |
| SES -> P. A | -0.019 (0.013) | 1.422 (0.149) |
| SES -> Unhealthy food stores | 0.065 (0.046) | 1.407 (0.155) |
| Unhealthy food stores -> DQI | -0.002 (0.016) | 0.098 (0.923) |
